# Incidence of Traumatic Brain Injury in a Longitudinal Cohort of Older Adults

**DOI:** 10.1001/jamanetworkopen.2024.14223

**Published:** 2024-05-31

**Authors:** Erica Kornblith, L. Grisell Diaz-Ramirez, Kristine Yaffe, W. John Boscardin, Raquel C. Gardner

**Affiliations:** 1 San Francisco Veterans Affairs Health Care System, San Francisco, California; 2 Department of Psychiatry, University of California San Francisco, San Francisco; 3 Department of Medicine, University of California, San Francisco, San Francisco; 4 Northern California Institute for Research and Education, San Francisco, California; 5 Department of Neurology, University of California, San Francisco, San Francisco; 6 Department of Epidemiology and Biostatistics, University of California, San Francisco, San Francisco; 7 Joseph Sagol Neuroscience Center, Sheba Medical Center, Ramat Gan, Israel

## Abstract

**Importance:**

Traumatic brain injury (TBI) occurs at the highest rate in older adulthood and increases risk for cognitive impairment and dementia.

**Objectives:**

To update existing TBI surveillance data to capture nonhospital settings and to explore how social determinants of health (SDOH) are associated with TBI incidence among older adults.

**Design, Setting, and Participants:**

This nationally representative longitudinal cohort study assessed participants for 18 years, from August 2000 through December 2018, using data from the Health and Retirement Study (HRS) and linked Medicare claims dates. Analyses were completed August 9 through December 12, 2022. Participants were 65 years of age or older in the HRS with survey data linked to Medicare without a TBI prior to HRS enrollment. They were community dwelling at enrollment but were retained in HRS if they were later institutionalized.

**Exposures:**

Baseline demographic, cognitive, medical, and SDOH information from HRS.

**Main Outcomes and Measures:**

Incident TBI was defined using inpatient and outpatient *International Classification of Disease*s, *Ninth* or *Tenth Revision,* diagnosis codes received the same day or within 1 day as the emergency department (ED) visit code and the computed tomography (CT) or magnetic resonance imaging (MRI) code, after baseline HRS interview. A cohort with TBI codes but no ED visit or CT or MRI scan was derived to capture diagnoses in nonhospital settings. Descriptive statistics and bivariate associations of TBI with demographic and SDOH characteristics used sample weights. Fine-Gray regression models estimated associations between covariates and TBI, with death as a competing risk. Imputation considering outcome and complex survey design was performed by race and ethnicity, sex, education level, and Area Deprivation Index percentiles 1, 50, and 100. Other exposure variables were fixed at their weighted means.

**Results:**

Among 9239 eligible respondents, 5258 (57.7%) were female and 1210 (9.1%) were Black, 574 (4.7%) were Hispanic, and 7297 (84.4%) were White. Mean (SD) baseline age was 75.2 (8.0) years. During follow-up (18 years), 797 (8.9%) of respondents received an incident TBI diagnosis with an ED visit and a CT code within 1 day, 964 (10.2%) received an incident TBI diagnosis and an ED code, and 1148 (12.9%) received a TBI code with or without an ED visit and CT scan code. Compared with respondents without incident TBI, respondents with TBI were more likely to be female (absolute difference, 7.0 [95% CI, 3.3-10.8]; *P* < .001) and White (absolute difference, 5.1 [95% CI, 2.8-7.4]; *P* < .001), have normal cognition (vs cognitive impairment or dementia; absolute difference, 6.1 [95% CI, 2.8-9.3]; *P* = .001), higher education (absolute difference, 3.8 [95% CI, 0.9-6.7]; *P* < .001), and wealth (absolute difference, 6.5 [95% CI, 2.3-10.7]; *P* = .01), and be without baseline lung disease (absolute difference, 5.1 [95% CI, 3.0-7.2]; *P* < .001) or functional impairment (absolute difference, 3.3 [95% CI, 0.4-6.1]; *P* = .03). In adjusted multivariate models, lower education (subdistribution hazard ratio [SHR], 0.73 [95% CI, 0.57-0.94]; *P* = .01), Black race (SHR, 0.61 [95% CI, 0.46-0.80]; *P* < .001), area deprivation index national rank (SHR 1.00 [95% CI 0.99-1.00]; *P* = .009), and male sex (SHR, 0.73 [95% CI, 0.56-0.94]; *P* = .02) were associated with membership in the group without TBI. Sensitivity analyses using a broader definition of TBI yielded similar results.

**Conclusions and Relevance:**

In this longitudinal cohort study of older adults, almost 13% experienced incident TBI during the 18-year study period. For older adults who seek care for TBI, race and ethnicity, sex, and SDOH factors may be associated with incidence of TBI, seeking medical attention for TBI in older adulthood, or both.

## Key Points

**Question:**

How common is traumatic brain injury (TBI) among older adults in the US?

**Findings:**

Over an 18-year study period, this cohort study found that 12.9% of 9239 study respondents experienced TBI. Race and ethnicity, sex, cognition, educational level, and medical conditions were associated with TBI status.

**Meaning:**

Findings of this study suggest that incident TBI is common among older adults and may be associated with demographic or social factors.

## Teaser

This cohort study updates existing traumatic brain injury surveillance data to capture nonhospital settings and to explore whether social determinants of health are associated with traumatic brain injury incidence among older adults.

## Introduction

Traumatic brain injury (TBI) is common in the US and occurs at the highest rate in older adulthood.^1,2^ It is associated with several negative cognitive and functional outcomes and staggering health care costs.^3^ Vulnerability to TBI may differ by sociodemographic factors and presence of cognitive impairment or dementia,^3^ yet TBI incidence among older adults and their subgroups is poorly characterized. The most recent comprehensive numbers, from 2014, come from a study that included only hospital codes and as such do not capture older adults who receive treatment for a TBI in an outpatient (ie, primary care, urgent care) setting.^4^

Social determinants of health (SDOH) are defined as the conditions in which individuals exist in their daily lives, which are shaped by the distribution of resources and presence of obstacles at both the global and local levels.^5^ These social, demographic, and contextual factors may include education and employment, socioeconomic status, region, and community or neighborhood variables. Because they reflect the day-to-day outcomes of historical legacies of systemic oppression, SDOH are highly correlated with race and ethnicity and may underly race- and ethnicity-based health disparities. Social determinants of health impact risk for multiple medical conditions and are thought to underly disparities in conditions such as diabetes and cardiovascular disease,^6^ which are associated with increased risk of TBI^7^ as well as with accelerated aging and dementia.^8^ However, the impact of SDOH on risk of TBI is unknown. Social determinants of health also impact how individuals interact with the medical system and whether and how they access care for a particular injury or illness.

By accessing linked Medicare claims data to obtain incident TBI diagnoses, we were able to leverage the detailed cognitive, demographic, and SDOH information available in the nationally representative Health and Retirement Study (HRS) to explore the association of cognitive status, demographics, and SDOH with the incidence of TBI in a diverse cohort of older adults. We were also able to further understanding of the epidemiology of TBI among older adults by including data up to 2018 (compared with the most recent comprehensive data, from 2014). Moreover, we included codes received in nonhospital settings (ie, outpatient, primary care, urgent care, and specialty care), thereby adding granularity to our understanding of risk for TBI among older adults. Although the use of diagnostic codes has limitations, the current work provides opportunities to examine both the scale of TBI as a medical issue for older adults as well as to document potential biases in access to care or documentation of TBI diagnoses.

Our goal was to investigate the association of demographics and SDOH with TBI incidence to inform both targeted intervention (eg, primary TBI prevention) for groups most at risk and to explore identification and mollification of relevant structural and contextual factors to reduce risk of TBI for older adults.

## Methods

This cohort study follows the Strengthening The Reporting of Observational Studies in Epidemiology (STROBE) reporting guideline. All study procedures were approved by institutional review boards and the University of California, San Francisco, and San Francisco Veterans Affairs Medical Center. The requirement to obtain informed consent was waived by these entities because of the use of deidentified archival data.

### Participants

**Figure 1.**
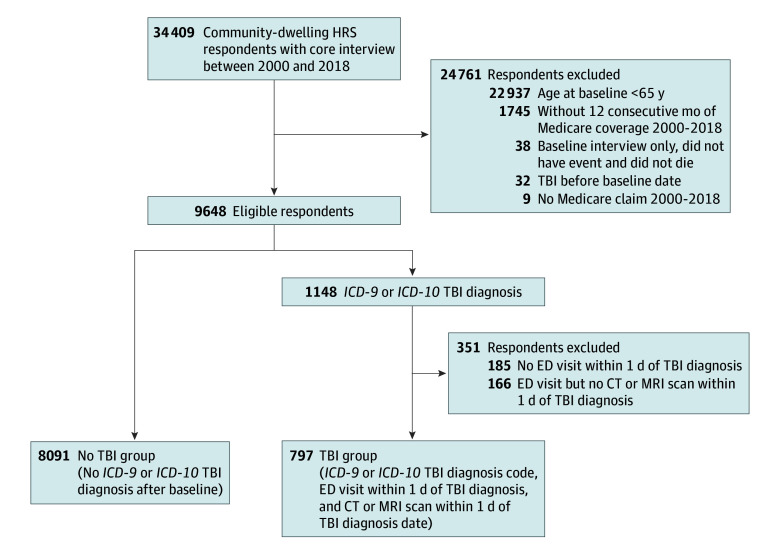
Cohort Derivation CT indicates computed tomography; ED, emergency department; HRS, Health and Retirement Study; *ICD-9* or *ICD-10*, *International Classification of Diseases, Ninth* or *Tenth Revision;* MRI, magnetic resonance imaging; and TBI, traumatic brain injury.

From 34 409 HRS community-dwelling age-eligible respondents with a core interview in 2000 or later, we created a nationally representative cohort of 9239 community-dwelling seniors enrolled in the HRS in survey waves of 2000 through 2018 who had Medicare-linked data from 2000 through 2018 (latest year available as of December 1, 2023). Respondents were community dwelling at enrollment but were retained in HRS if they were later institutionalized. See Figure 1 for details of exclusion and cohort derivation. The baseline date was the date of the first age-eligible HRS core interview in the community in 2000 or later. The HRS is sponsored by the National Institute on Aging and is conducted by the University of Michigan.^9,10^

### Outcome

We identified incident TBI in inpatient and outpatient Medicare 2000 through 2018 claims. To ascertain TBI status, we used an existing, comprehensive, and updated list of *International Classification of Diseases, Ninth Revision* (*ICD-9*) and *Tenth Revision* (*ICD-10*) diagnosis codes developed by the Defense and Veterans Brain Injury Center and the Armed Forces Health Surveillance Branch for TBI surveillance (2016 criteria) to identify incident TBI in Medicare data, received the same day or 1 day before or after an emergency department (ED) visit code and a computed tomography (CT) or magnetic resonance imaging (MRI) scan code occurring after the enrollee’s baseline HRS interview. To conduct a sensitivity analysis, we also derived a cohort of older adults who received a TBI code but no ED visit or CT or MRI scan to capture individuals receiving diagnoses in nonhospital settings. The time to first event was computed as the time difference between the baseline date and “claim admission date” (inpatient file) or “claim from date” (outpatient file) from the first claim with a TBI diagnosis code.

### Exposures

We examined the following 2 exposures. The first exposure was cognitive status as assessed using the Langa-Weir dementia probability, a predictive model, frequently used in dementia research and well-validated,^11^ which classifies each HRS participant as having Alzheimer disease or related dementia, cognitive impairment with no dementia, or normal cognition. The second exposure was demographic and SDOH variables. The HRS includes self-reported data on demographics (race and ethnicity and sex), detailed information about educational level, employment, and individual- and neighborhood-level socioeconomic status as well as data on rurality. The categories for race and ethnicity included Black, Hispanic, White, or other. Education was defined as a 4-level variable: less than high school or General Educational Development, high school, some college, or college and above. Employment was defined as a dichotomous variable based on self-report of whether the respondent was currently employed for pay at the time of baseline survey. Neighborhood socioeconomic status was defined by the national percentile of the block group area deprivation index national rank^12^ (ADI) score (range 1-100, with higher scores indicating higher levels of deprivation). Individual socioeconomic status was defined by weighted quartiles of total assets: $46 879 or lower, more than $46 879 to $154 969, more than $154 969 to $387 837, more than $387 837. Rurality was a harmonized, dichotomous variable reflecting urban vs rural residence at the time of entry into HRS.

#### Adjustment Variables

We adjusted analyses for splines of age (defined at Harrell default quantiles of age with 4 knots at 65.7, 70.9, 77.6, and 88.5) as well as important medical and behavioral factors (cardiovascular disease, splines of body mass index [BMI], alcohol use, stroke, diabetes, and lung disease); any activity of daily living difficulty in bathing, dressing, toileting, transferring, and eating; marital status; veteran status; physical activity (a dichotomous variable, with participants coded as 1 [yes] if they reported performing vigorous physical activity more than once per week); ADI score; and continuous format psychiatric symptom severity (ie, Center for Epidemiologic Studies Depression Scale score) variables associated with, and that may confound, the association between various exposures and incident TBI. All covariates were ascertained at the time of entry into the study, that is, the date of the respondent’s first age-eligible HRS core interview in the community in 2000 or later.

### Statistical Analysis

We accounted for the HRS complex survey design. For continuous variables, the assumption of normality was checked by visual examination using histograms with a normal-density curve and quantile-quantile plots. We used Fine-Gray competing risks regression^13^ to estimate the associations between exposures and TBI in the presence of death as a competing risk. We used HRS survey weights and the robust sandwich variance estimation with a linear combination of the number of clusters and strata.

#### Residuals and Diagnostic Measures

We checked the proportional hazards assumption by visual examination. We also tested the interaction terms with age, BMI, and ADI. Overall, the plotted curves for each level of the categorical exposure variables in the model were approximately parallel. Moreover, the inclusion of the interaction terms with time of age, BMI, and ADI did not meaningfully affect the results. Thus, the proportional hazards assumption was not violated.

#### Multiple Imputation

We did not impute ADI since the algorithm to compute this variable includes several variables that were not present in our dataset. For the rest of the exposure variables, we imputed missing values using the fully conditional specification method. For most of the exposure variables, except for race and ethnicity and BMI, we used the logistic regression method with the default binary logit model for 8 binary predictors and the cumulative logit function for the ordered categorical variable education. For race and ethnicity, we used the discriminant function method, and for BMI we used the predictive mean matching method. Imputation was performed considering the outcome and complex survey design variables, including survey weights, clusters, and strata.

We computed the mean cumulative incidence across 50 multiple imputed datasets by race and ethnicity, sex, educational level, and ADI percentiles 1, 50, and 100. We fixed the rest of the exposure variables at their weighted means.

All analyses were performed with SAS/STAT, version 15.2 (SAS Institute Inc), Stata, version 17.0 (StataCorp LLC), and R, version 4.2.1 (R Project for Statistical Computing). A 2-sided value of *P* < .05 was considered statistically significant.

## Results

The final analytic cohort included 9239 older adults (5258 [57.7%] female, 3981 [42.3%] male; 1210 [9.1%] Black, 574 [4.7%] Hispanic, 7197 [84.4%] White, and 152 [1.8%] self-identified as American Indian or Alaskan Native; Asian, Native Hawaiian, or Other Pacific Islander; or other, more than one, or unknown race). Mean (SD) baseline age was 75.2 (8.0) years. Bivariate associations between exposures and TBI incidence are shown in Table 1. Respondents were most likely to have less than a high school education and to reside in urban areas of the southern US. During the study follow-up period of approximately 18 years, 797 (8.9%) received an incident TBI diagnosis with an ED and a CT code, 964 (10.2%) received ED treatment for an incident TBI, and 1148 (12.9%) received an incident TBI diagnosis code either with or without ED and CT. The percentage of respondents missing at least 1 exposure variable in the primary model was 14.3%, with 10.4% missing Center for Epidemiologic Studies Depression Scale score, 2.7% missing ADI score, and 1.3% missing BMI. The remaining covariates had less than 1% missing.

**Table 1.** Baseline Characteristics of HRS Enrollees With or Without TBI During Study Follow-Up^a^

Characteristic	Enrollees, No. (%) (N = 9239)	Enrollees Without TBI, No. (%) (n = 8442)	Enrollees With TBI, No. (%) (n = 797)	*P* value
**Demographics or SDOH**				
Age, mean (SD) y	75.2 (8.0)	75.2 (8.1)	75.2 (7.3)	.94
Sex				
Female	5258 (57.7)	4756 (57.1)	502 (64.2)	<.001
Male	3981 (42.3)	1723 (43.3)	1393 (35.0)	
Race and ethnicity				
Black	1210 (9.1)	1148 (9.5)	62 (5.4)	<.001
Hispanic	574 (4.7)	534 (4.9)	40 (3.5)	
White	7297 (84.4)	6617 (83.9)	680 (89.1)	
Other^b^	152 (1.8)	NR (1.7)^c^	NR (2.1)^c^	
Educational level				
<HS or GED	3357 (34.5)	3132 (35.3)	225 (26.5)	<.001
HS	2880 (32.0)	2605 (31.7)	275 (35.9)	
Some college	1576 (17.4)	1433 (17.3)	143 (18.1)	
≥College degree	1425 (16.1)	1271 (15.8)	154 (19.6)	
Married or partnered	5530 (57.2)	5036 (57.0)	494 (59.1)	.36
Working for pay	1552 (17.0)	1143 (16.9)	139 (17.9)	.55
Total wealth, $				
≤46 879	2443 (25.0)	2263 (25.3)	180 (22.2)	.006
>46 879-154 969	2314 (24.9)	2162 (25.2)	179 (21.5)	
>154 969-387 837	2230 (25.1)	2026 (25.1)	204 (25.4)	
>387 837	2225 (25.0)	1991 (24.4)	234 (30.9)	
Region of residence				
Northeast	1517 (18.2)	1369 (17.9)	148 (20.9)	.41
Midwest	2573 (29.3)	2349 (29.5)	224 (27.8)	
South	3925 (37.8)	3608 (37.9)	317 (36.8)	
West	1215 (14.6)	1107 (14.6)	108 (14.5)	
Rural residence	2927 (33.7)	2700 (34.0)	227 (30.4)	.08
Veteran status	2641 (28.5)	2436 (28.8)	205 (25.4)	.06
Total wealth (includes secondary residence) at baseline, mean (SD), $	375 606.0 (936 308.4)	370 176.3 (938 226.1)	431 012.0 (914 320.7)	.06
Area Deprivation Index, mean (SD)^d^	56.91 (1.6)	58.0 (31.7)	53.3 (30.7)	<.001
**Baseline medical characteristics**				
High blood pressure	4863 (52.0)	4441 (52.0)	422 (52.3)	.84
Stroke	976 (10.3)	896 (10.4)	80 (10.1)	.85
Diabetes	1546 (16.1)	1425 (16.2)	121 (15.6)	.65
Lung disease	857 (9.3)	820 (9.8)	37 (4.7)	<.001
Cardiovascular disease	2706 (29.2)	2491 (29.4)	215 (27.2)	.23
Problems with ADL	1839 (19.6)	1669 (19.9)	140 (16.6)	.03
BMI				
Mean (SD) (n = 9123)	26.3 (5.6)	26.3 (5.6)	26.4 (5.7)	.73
<18.5	247 (2.7)	NR (2.8%)^c^	NR (1.6%)^c^	.12
18.5 to <25	3448 (38.4)	3132 (38.2)	316 (40.5)	
25 to <30	3600 (39.4)	3309 (39.7)	291 (37.0)	
≥30	1828 (19.5)	1663 (19.3)	165 (20.9)	
Presence of depressive symptoms	2046 (24.4)	1881 (24.6)	165 (22.6)	.25
Presence of alcohol use	3762 (42.0)	3417 (41.9)	345 (43.3)	.42
Baseline cognition				
Normal	6197 (68.4)	5616 (67.8)	581 (73.9)	.001
CIND	2105 (22.3)	1949 (22.6)	156 (19.0)	
Dementia	937 (9.4)	877 (9.6)	60 (7.1)	
Proxy interview	959 (9.7)	891 (9.9)	68 (7.6)	.07
Died during follow-up	7033 (76.2)	6409 (76.0)	624 (78.1)	.22
Imputed memory score at baseline, mean (SD) (n = 9228)	0.7 (0.7)	0.7 (0.7)	0.8 (0.7)	<.001
Imputed dementia probability at baseline, mean (SD) (n = 9224)	0.1 (0.3)	0.1 (0.3)	0.1 (0.2)	.01
Total cognition summary score at baseline, range 0-35, mean (SD) (n = 8267)	21.6 (6.0)	21.6 (6.0)	22.1 (5.6)	.02

Abbreviations: ADL, activities of daily living; BMI, body mass index (calculated as weight in kilograms divided by height in meters squared); CIND, cognitive impairment with no dementia; GED, General Educational Development; HRS, Health and Retirement Study; HS, high school; NR, not reported; SDOH, social determinants of health; TBI, traumatic brain injury.

^a^ All analyses account for complex survey weights, clusters, and strata.

^b^ Category includes respondents who identified their race or ethnicity as Alaskan Native, American Indian, Asian, Native Hawaiian, Pacific Islander, or other.

^c^ Cell numbers below 25 cannot be reported due to restricted data.

^d^ Area Deprivation Index national rank scores range from 0 to 100, with higher values indicating higher level of deprivation.

Table 1 shows medical, demographic, and SDOH characteristics of the sample with (n = 797) or without (n = 8442) TBI. Older adults who experienced incident TBI during the study period were more likely to be female (absolute difference, 7.0 [95% CI, 3.3-10.8]; *P* < .001) and White (absolute difference, 5.1 [95% CI, 2.8-7.4]; *P* < .001), have normal cognition (vs cognitive impairment or dementia; absolute difference, 6.1 [95% CI, 2.8-9.3]; *P* = .001), higher education (absolute difference, 3.8 [95% CI, 0.9-6.7]; *P* < .001), and wealth (absolute difference, 6.5 [95% CI, 2.3-10.7]; *P* = .01), and be without baseline lung disease (absolute difference, 5.1 [95% CI, 3.0-7.2]; *P* < .001) or functional impairment (absolute difference, 3.3 [95% CI, 0.4-6.1]; *P* = .03). Respondents who endorsed US military veteran status were less likely to experience an incident TBI (absolute difference, 3.5 [95% CI, 0.1-7.0]; *P* = .06) as well.

Sensitivity analyses including elders who received an incident TBI code but no ED visit and CT or MRI scan (n = 1148) showed similar results (eTables 1 and 2 in Supplement 1). Other sensitivity analyses using codes only (ie, TBI diagnosis but no ED visit or imaging scan) showed similar results when stratified by hospital admission status, with the exception that female sex was no longer associated with incident TBI (HR, 0.81 [95% CI, 0.52-1.27]; *P* = .36) (eTable 3 in Supplement 1). In additional sensitivity analyses stratified by public vs private or other hospital cluster, the findings for sex (HR, 0.82 [95% CI, 0.55-1.23]; *P* = .35), race and ethnicity (HR, 0.67 [95% CI, 0.33-1.37]; *P* = .27) and educational level (HR, 0.59 [95% CI, 0.34-1.03]; *P* = .06) no longer reached statistical significance. However, the sample number was much smaller due to missing data on hospital cluster 2000 through 2009, resulting in the exclusion of several covariates from the model. Thus, these results should be interpreted with caution (eTable 4 in Supplement 1).

**Figure 2.**
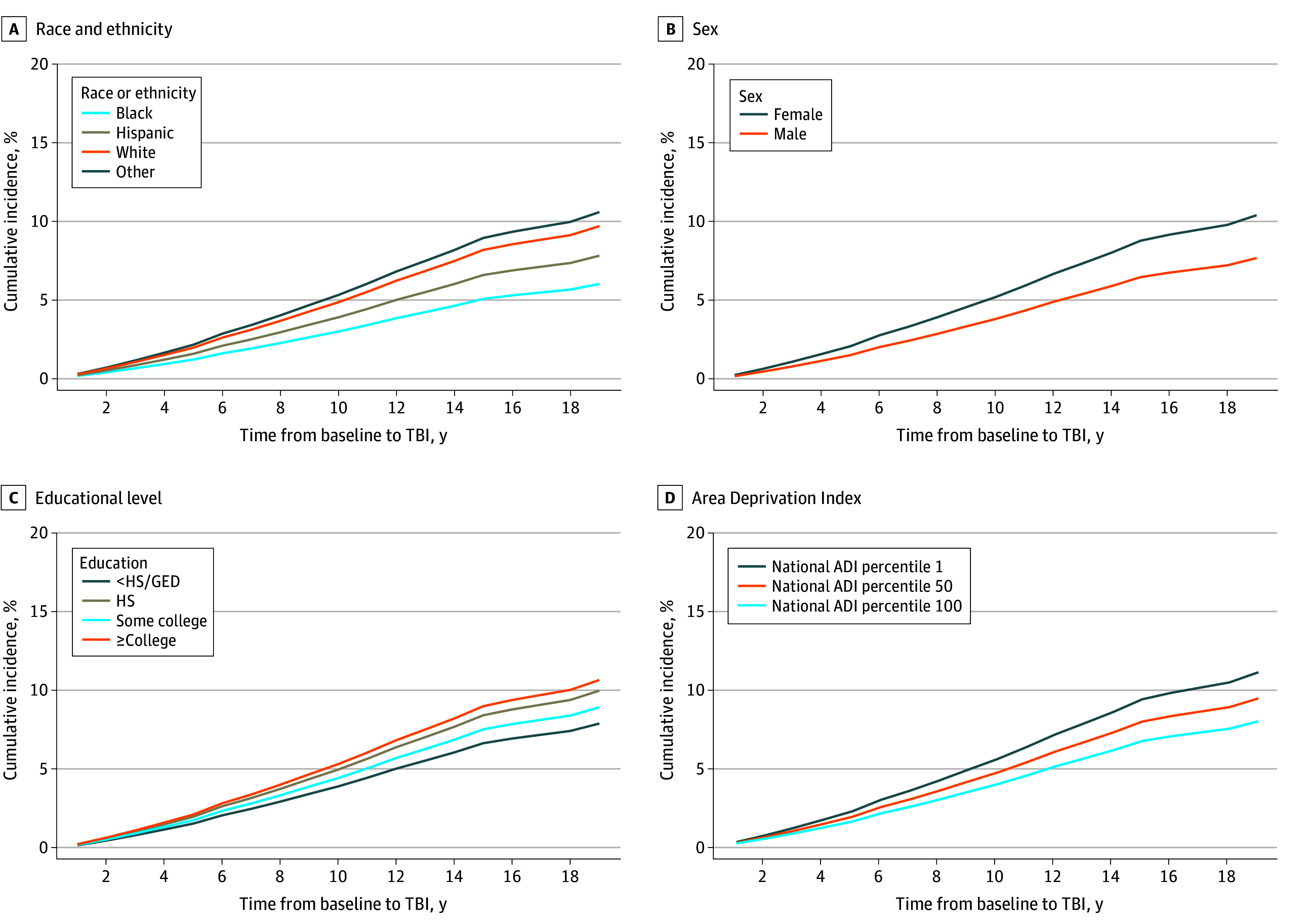
Adjusted Imputed Cumulative Incidence of TBI, Accounting for Mortality by Race and Ethnicity, Sex, Educational Level, and Area Deprivation Index Percentile (ADI) Data were adjusted for demographic and health characteristics. ADI score range is 1 to 100, with higher scores indicating higher levels of deprivation. GED indicates General Educational Development; HS, high school; and TBI, traumatic brain injury.

Figure 2 shows imputed cumulative incidence of TBI over time by race or ethnicity, sex, educational level, and neighborhood characteristics (ie, ADI^12^). Imputation considering outcome and complex survey design was performed by race and ethnicity, sex, education level, and ADI percentiles 1, 50, and 100. Other exposure variables were fixed at their weighted means. Female sex, higher education, and residence in higher resource areas were associated with higher incidence of TBI. Examining the plots for race and ethnicity, we found that cumulative incidence of TBI was highest for respondents who categorize their race and ethnicity as *other*. White race was associated with higher cumulative incidence compared with Black and Hispanic race and ethnicity. Notably, the change in trajectory around 2015 is most likely due to the change from using *ICD-9* to *ICD-10* codes and not representative of a true change in TBI incidence. The increase in trajectory slope around 2018 may be associated with providers experiencing increased comfort in the use of the *ICD-10* codes.

In multivariate models with incident TBI as the outcome and adjusted for demographics and medical and SDOH factors, lower educational level (subdistribution HR [SHR], 0.73 [95% CI, 0.57-0.94]; *P* = .01), Black race (SHR, 0.61 [95% CI, 0.46-0.80]; *P* < .001), and male sex (SHR, 0.73 [95% CI, 0.56-0.94]; *P* = .02) were associated with lower rates of incident TBI (Table 2). Specifically, White race was associated with elevated incidence of TBI compared with respondents identifying as Black or Hispanic race and ethnicity, and Black race was associated with a 40% decrease in the subdistribution hazard of TBI (SHR, 0.61 [95% CI, 0.44-0.85]; *P* < .01).

**Table 2.** Multivariate Models Showing Time to Incident TBI^a^^,^^b^

Parameter	SHR (95% CI)	*P* value
Sex		
Female	1 [Reference]	.02
Male	0.73 (0.56-0.94)	
Race and ethnicity		
Black	0.61 (0.46-0.80)	<.001
Hispanic	0.80 (0.55-1.16)	.24
White	1 [Reference]	NA
Other^c^	1.10 (0.06-1.85)	.73
High blood pressure	1.07 (0.93-1.24)	.34
Stroke	1.14 (0.87-1.48)	.34
Diabetes	1.05 (0.84-1.32)	.67
Lung disease	0.52 (0.36-0.76)	.001
Cardiovascular disease	1.02 (0.86-1.21)	.84
Functional impairment	0.89 (0.71-1.10)	.28
Depressive symptoms	0.96 (0.79-1.16)	.68
Alcohol use	0.93 (0.77-1.12)	.44
Cognition		
Normal	1 [Reference]	NA
CIND	0.98 (0.80-1.22)	.89
Dementia	0.93 (0.69-1.26)	.94
Educational level		
<HS/GED	0.73 (0.57-0.94)	.01
HS	0.93 (0.75-1.16)	.53
Some college	0.83 (0.64-1.08)	.16
≥College	1 [Reference]	NA
Marital status		
Married or partnered	1 [Reference]	.12
Not married	0.87 (0.73-1.04)	
Employment status		
Working	1 [Reference]	.15
Not working	1.11 (0.91-1.35)	
Rurality		
Urban	1 [Reference]	.51
Rural	0.93 (0.76-1.15)	
Veteran status		
Yes	0.94 (0.72-1.26)	.71
No	1 [Reference]	
Total assets, weighted quartile		
≤$46 879	0.99 (0.75-1.31)	.95
>$46 879-$154 969	0.84 (0.68-1.03)	.09
>$154 969-$387 837	0.85 (0.68-1.04)	.12
>$387 837	1 [Reference]	NA
Physically active >1/w		
Yes	0.88 (0.74-1.04)	.13
No	1 [Reference]	
Area Deprivation Index National Rank	1.00 (0.99-1.00)	.009
Age^d^		
Age (linear)	1.10 (1.02-1.18)	.009
Age (restricted cubic spline 1)	0.76 (0.54-1.07)	.12
Age (restricted cubic spline 2)	1.58 (0.75-3.30)	.23
BMI^e^		
BMI (linear)	0.98 (0.92-1.04)	.43
BMI (restricted cubic spline 1)	1.12 (0.89-1.42)	.33
BMI (restricted cubic spline 2)	0.72 (0.33-1.55)	.40

Abbreviations: BMI, body mass index; CIND, cognitive impairment no dementia; GED, General Educational Development; HS, high school; NA, not applicable; SHR, subdistribution hazard ratio; TBI, traumatic brain injury.

^a^ Number of respondents, 8991; number of events, 781.

^b^ Models were adjusted for demographics and medical and social determinants of health factors. All analyses account for complex survey weights, clusters, and strata.

^c^ Category includes respondents who identified their race as Alaskan Native, American Indian, Asian, Native Hawaiian, Pacific Islander, or other.

^d^ Splines of age were defined at Harrell default quantiles of age with 4 knots at 65.7, 70.9, 77.6, and 88.5.

^e^ Splines of BMI were defined at Harrell default quantiles of BMI with 4 knots at 19.4, 24.3, 27.5, and 35.3.

## Discussion

The results of this population-based cohort study assessing the incidence of TBI and factors associated with incident TBI among older adults in the US indicated an extremely high rate of TBI in this group. This finding is consistent with other recent work documenting the scope of the problem of TBI in older adults.^2^ As the US population rapidly ages, the epidemiology of TBI is changing, and older adults are the age group most likely to be hospitalized or die from TBI.^2^ However, little is known about which older adults are most vulnerable to incident TBI. This information is important from a public health perspective because TBI increases risk of multiple negative outcomes associated with aging, including multisystem (neurologic, cardiovascular, and endocrine) medical comorbidity,^14^ loss of functional independence^15^ and reduced quality of life.^16^ Our work suggests associations of TBI incidence with race and ethnicity, sex, and demographic factors, with highest incidence possibly associated with healthy, active, high-socioeconomic status White women.

Whereas strong evidence for race-based and regional disparities in TBI-related deaths exist,^16^ incidence of TBI is harder to quantify because of issues with reporting and differences in public health messaging around treatment seeking, as well as differences in access to care, which vary by demographics and SDOH. Our work used Medicare claims data to identify incident TBI. Although evidence does exist that claims data have excellent criterion validity,^17^ it is also true that limitations around confidence in the ascertainment of TBI diagnoses that may be related to access to health care, diagnostic bias, or other factors are unavoidable. Prospective studies featuring a comprehensive diagnostic evaluation are the gold standard in TBI research. However, even existing prospective studies have similar problems with respect to being able to enroll only individuals who seek care and consent to participation, which may limit the diversity of cohorts. Self-reported data may capture more of the true breadth and differential prevalence of TBI diagnoses but also can be unreliable regarding presence, severity, and outcomes of the condition being studied.^18^ Given these methodological challenges, documentation and awareness of detection bias in TBI research and care are important for designing future studies and increasing both the accuracy of scientific knowledge and the quality and equity of health care.

We aimed to circumvent some of these methodological challenges in the current work by utilizing a large, nationally representative survey dataset in which most participants agreed to have their responses linked to Medicare claims data. Thus, we were able to examine incidence of TBI adjusted for multiple important demographic and structural variables, such as the ADI, an objective measure of neighborhood resource deprivation that has been associated with a wide array of medical and psychosocial outcomes.^12^ Our results also update the existing information available on nationwide TBI incidence (ED visits, hospitalizations, and deaths; more recent data up to 2020 exist on hospital admissions and deaths^16^) in older adults, which covers up to 2014^19^; our work covers 2000 through 2018 and also includes both *ICD-9* and *ICD-10* diagnosis codes. We were also able to show similar results for definitions of TBI with or without hospital treatment (ie, ED code and CT or MRI scan), thereby addressing a limitation of existing older adult TBI surveillance by showing differential incidence of TBI that includes nonhospital settings, such as primary care, urgent care, and specialty care.^20^

Our results suggest that almost 13% of US older adults received treatment for an incident TBI experienced during the 18-year study period, and that race, sex, and SDOH factors may be associated with incident TBI. In this diverse sample of older Americans, our also work suggested that an increased rate of TBI was associated with healthy, wealthy, White female individuals. This finding stands in contrast to existing understanding of factors associated with incident TBI, specifically, male sex has long been considered a risk factor for TBI,^21^ but our work suggests this may not hold for the older adult population. Prior work documenting that although older men are at higher risk for head injury, women have worse outcomes^22^ suggests that our results showing increased incidence of TBI in older women may reflect more immediate negative outcomes leading to higher rates of care seeking. Since the most recent surveillance data available show higher rates of hospitalization and death associated with TBI for older men, as well as higher rates of the most common mechanisms of injury (falls, motor vehicle crashes),^2,19^ perhaps older women are more likely to experience a less serious head injury and not be admitted to a hospital. Interestingly, however, these results are consistent with prior work from our group suggesting the possibility of a greater prevalence of TBI in older female veterans compared with male veterans in a nationwide Veterans Affairs Health Care System dataset^23^ and with a recent study by Yashkin and colleagues^24^ using the HRS cohort to show higher incidence of TBI for older women compared with men. Sex differences in geriatric TBI is an area with interesting opportunities for further study.

Many medical conditions are more common in individuals and populations with lower education and financial resources. Our findings suggesting an increased rate of TBI in wealthier respondents, therefore, run counter to expectations and raise important questions about whether this finding reflects a real difference in TBI incidence or, because we were able to study only incident head injuries for which individuals received care, a difference in access to or willingness to seek care. An older study, published in 2007,^25^ found that 42% of respondents to an online survey did not seek medical care after experiencing a TBI, and older respondents were less likely to seek care as well as those experiencing a mild TBI and those who were injured at home. Of the 1381 survey respondents with TBI, 584 (42%) did not seek medical care. A similar study found that 50% of adults who experience what they suspect is a TBI do not seek medical care, and most of these injuries were related to falls.^26^ Thus, older adults who experience falls, the largest segment of US citizens experiencing incident TBI, are also the least likely to seek care. In addition, lower resourced individuals may be even less likely to seek care due to multiple factors, including but not limited to the racial and ethnic microaggressions that commonly occur in the medical setting.^27^ Thus, our estimates of TBI incidence among US older adults are likely to be lower than the true burden of TBI in this population and may not reflect true differential incidence of TBI based on race and ethnicity and other demographics.

In contrast, it is possible that our findings reflect that older adults who are healthier, wealthier, and more active are more able or likely to engage in activities, such as skiing or horseback riding, that carry risk for TBI. An additional alternative explanation is bias (intentional or unintentional) on the part of health care providers who may be more likely to diagnose a TBI in healthy, wealthy, white women who present to the ED after a fall. Indeed, recent work by Tsoy and colleagues^28^ using a large representative sample of Medicare beneficiaries in California shows Black-, Asian-, and Hispanic-identified older adults have delayed and less comprehensive dementia diagnoses in claims data. A similar association (ie, reduced comprehensiveness of assessment and diagnosis) may also be true for TBI and may bias our results. However, we preformed sensitivity analyses showing that respondents with a TBI code but no brain imaging or ED visit were similarly distributed across race and ethnic groups compared with the sample as a whole. Other sensitivity analyses showed a similar pattern of results in participants who were admitted to the hospital vs those who were not and in public vs private hospitals, although because of limitations in our available data, the impact of hospital admission and cluster type may still bear further study.

We also identified differences in incident TBI based on cognitive status when first joining the HRS cohort: respondents who had cognitive problems may be less likely to experience incident TBI. Although this association was only found in bivariate analyses, this finding nevertheless runs counter to findings suggesting that individuals with cognitive impairment are more likely to fall and sustain a head injury. It is possible that respondents with cognitive impairment are more limited in their activity and therefore experience less opportunity to fall. But respondents with both milder cognitive impairment and frank dementia had lower incidence of TBI, and those with milder cognitive problems would not be suspected to experience significant activity limitations. Further work is needed to understand the impact of cognitive status on incident TBI over time.

A recent analysis of a large community-based cohort of older adults followed up for more than a mean of 20 years shows that older adults with a previous head injury are much more likely to fall again.^29^ Although we did not measure lifetime history of TBI, and respondents with a prevalent TBI at baseline were excluded, there were only 32 respondents excluded for this reason. While our current study does not address the impact of multiple TBIs, our work defining a cohort of respondents experiencing incident TBI allows for future follow-up study of respondents who experienced incident TBI to examine rates of subsequent falls and head injuries and their impact on outcomes.

In our population-based cohort, we showed that older veterans had lower rates of incident TBI during the study period, a finding consistent with our group’s earlier work documenting that nonveterans in the HRS sample were more likely to have a lifetime history of TBI.^30^ Although younger veterans are generally understood to be at higher risk of head injury compared with nonveterans because of combat and training-related exposures, the impact of age on TBI incidence for veterans remains unclear. Further research is needed to elucidate TBI rates in older veterans and nonveterans, incident TBI risk for aging veterans, and prevention strategies.

### Limitations

There are some important limitations to our study that may affect the interpretation and generalizability of our results. Receiving neuroimaging is the current standard of care for older adults who present to the ED after a possible TBI. However, this definition of TBI is conservative and may have caused us to miss cases. Still, sensitivity analyses in which we identified TBI cases without this requirement yielded similar results (eTables 1 and 2 in Supplement 1). Moreover, most sex, race and ethnicity, and other important SDOH factors, such as wealth and educational level, were based on self-report, and sex was coded as a binary variable only (ie, transgender individuals were not captured), likely excluding some of the true complexity of these variables. We also did not examine TBI severity in the current analysis. Furthermore, our sample had some limitations: we were unable to examine race and ethnicity with the granularity we would have preferred due to small sample sizes. Specifically, work examining incidence of TBI among older Asian and Native American individuals and other minoritized groups is urgently needed, and further research focused on minoritized older adult samples and those that identify their race as *other* would be helpful and provide insights for TBI treatment planning and prevention as this growing cohort of adults ages. Also, we used *ICD-9* and *ICD-10* codes in existing medical records to ascertain incident TBI. Although evidence does exist that claims data have excellent criterion validity,^17^ this method may result in less accurate categorization of participants compared with studies in which participants were given a comprehensive TBI screening.

## Conclusions

This novel cohort study assessing the association of demographics and SDOH with incident TBI in a nationwide sample includes updated data on incidence of TBI, including TBI treated outside a hospital setting, and suggests that older adults may experience differential incidence of TBI. However, it remains unclear to what extent demographics or access to care may affect who accesses treatment for TBI. Further research may lead to clarification of this question as well as to opportunities for both targeted intervention (eg, primary TBI prevention) for groups most at risk and identification and mollification of the most relevant structural and contextual factors (eg, access to care) to reduce incidence of TBI among older adults.

## Notes

Supplement 1. **eTable 1.** Baseline characteristics of HRS enrollees with and without TBI (diagnosis code only) during study follow-up **eTable 2.** Multivariate models showing time to incident TBI (diagnosis code only) **eTable 3.** Multivariate models showing time to incident TBI (diagnosis code only), stratified by hospital admission (inpatient vs outpatient) **eTable 4.** Multivariate models showing time to incident TBI (diagnosis code only), stratified by hospital cluster: private vs public or other

Supplement 2.
Data Sharing Statement
