# Supplementary material for: Incidence of Traumatic Brain Injury in a Longitudinal Cohort of Older Adults
Source: JAMA Netw Open. 2024 May 31;7(5):e2414223. doi: 10.1001/jamanetworkopen.2024.14223 (PMC11143459; doi:10.1001/jamanetworkopen.2024.14223)
Supplement: Supplement 2. — Data Sharing Statement [file jamanetwopen-e2414223-s002.pdf]

Data Sharing Statement

Kornblith. Incidence of Traumatic Brain Injury in a Longitudinal Cohort of Older Adults. *JAMA Netw Open*. Published May 31, 2024. doi:10.1001/jamanetworkopen.2024.14223

Data
Data available: No
